# Correction

**DOI:** 10.1093/sleepadvances/zpad010

**Published:** 2023-02-23

In the originally published versions of the below-listed manuscripts, an acknowledgement was missing: “This paper is part of the David F. Dinges Festschrift Collection. This collection is sponsored by Pulsar Informatics and the Department of Psychiatry in the Perelman School of Medicine at the University of Pennsylvania.”

This error has been corrected.

List of all affected DOI URLs:

https://doi.org/10.1093/sleepadvances/zpac034

https://doi.org/10.1093/sleepadvances/zpac040

